# Stability in flux: community structure in dynamic networks

**DOI:** 10.1098/rsif.2010.0524

**Published:** 2010-12-01

**Authors:** John Bryden, Sebastian Funk, Nicholas Geard, Seth Bullock, Vincent A. A. Jansen

**Affiliations:** 1 School of Biological Sciences, Royal Holloway, University of London, Egham TW20 0EX, UK; 2 Institute of Zoology, Zoological Society of London, Regent's Park, London NW1 4RY, UK; 3 School of Electronics and Computer Science, University of Southampton, Southampton SO17 1BJ, UK

**Keywords:** coevolutionary networks, opinion formation, modularity, dynamic equilibrium, protein–protein interaction

## Abstract

The structure of many biological, social and technological systems can usefully be described in terms of complex networks. Although often portrayed as fixed in time, such networks are inherently dynamic, as the edges that join nodes are cut and rewired, and nodes themselves update their states. Understanding the structure of these networks requires us to understand the dynamic processes that create, maintain and modify them. Here, we build upon existing models of coevolving networks to characterize how dynamic behaviour at the level of individual nodes generates stable aggregate behaviours. We focus particularly on the dynamics of groups of nodes formed endogenously by nodes that share similar properties (represented as node state) and demonstrate that, under certain conditions, network modularity based on state compares well with network modularity based on topology. We show that if nodes rewire their edges based on fixed node states, the network modularity reaches a stable equilibrium which we quantify analytically. Furthermore, if node state is not fixed, but can be adopted from neighbouring nodes, the distribution of group sizes reaches a dynamic equilibrium, which remains stable even as the composition and identity of the groups change. These results show that dynamic networks can maintain the stable community structure that has been observed in many social and biological systems.

## 1. Introduction

Many scenarios exist in nature and society where individuals or entities bias their interactions to a limited subset of a population. When populations split into subpopulations that interact strongly within themselves but much less strongly between themselves, they are said to exhibit community structure. In human and animal societies this means that they consist of partially independent groups, cliques and tribes [1–3], which can be important for studying epidemic spread [4]. This notion can be extended to more abstract representations of interactions in natural systems, such as in genetic, protein–protein and metabolic interaction networks that are structured into dynamic and functionally, spatially or temporally separated modules [5–7]; or in neural networks where neurons tend to cluster into groups based on activity patterns [8].

The analysis of networks using tools borrowed from graph theory has proved to be a useful approach for studying populations where individuals or entities within the population interact only with a certain subset of the remaining population, and significant effort has been put into developing methods to identify community structure in such networks [9–12]. The networks are usually taken to be static—they are presented or measured as snapshots in time, which neglects the fact that both the properties of individuals and the interactions between individuals will usually change over time. For example, human social and communication networks display complex community structure despite individuals continually changing their patterns of association [13]. Only recently has an increasing number of studies concentrated on the dynamical properties of networks [14], as well as their relevance to the spread of infectious diseases [15–19].

Previous models of dynamic networks have considered the coevolution of opinions and network connections under *homophily*—where edges are rewired to nodes of the same state [20]—and *heterophily*—where edges are rewired to nodes of a different state [21]. In these studies, homophilous processes are often contrasted with *state spread*—where states are transferred (or equilibrated) between nodes [21–26]. Existing work has tended to focus on exploring the probability of achieving consensus, or the time taken to do so, and has paid less attention to the dynamics that occur when multiple groups or communities coexist stably in the population.

Here, we focus on a topic that so far has received little attention: the emergence of community structure in dynamic networks. We introduce a model where each node has a state—which is either a fixed or dynamic property—and the network stays dynamic under homophilous and random rewiring. In addition to propagating states between nodes, we also use an ‘innovation’ process to continually introduce diversity into the population. With this model, we study the emergence and stability of community structure in the resulting dynamic networks, and how they relate to properties at either the level of individual nodes or at population level.

## 2. Methods

We first state our microscopic (individual-based) model as an algorithm. We will later study the corresponding macroscopic (population-level) model, which approximates the average behaviour of the microscopic model and allows for mathematical treatment of some aspects of the model behaviour.

We consider a network of *n* nodes and *m* undirected edges, where each node *i* is associated with a state *S*_*i*_ ∈ {*s*_1_, *s*_2_, *s*_3_, …}. We deliberately leave interpretations of the meaning of the state open at this point, as we will consider both scenarios where states are fixed properties of nodes and ones where they can spread over the edges of the network. Either way, what we deem states of nodes will form the basis for our implementation of homophilous rewiring, where nodes change edges to be preferentially connected to nodes of the same state.

In the individual-based model exactly one of the possible processes below, chosen with probability proportional to the corresponding rate, is invoked at each time step. The lengths of inter-event times are exponentially distributed, in line with [27], so that the time scale remains consistent across different parameter settings. Based largely on models of opinion flow [21] and of social group formation [28], we analyse the effects of two classes of simple processes on the network, one containing *rewiring* events and the other *state change* events. Let us first consider the class of processes dealing with rewiring: edges may either be rewired to nodes of the same state (homophilous rewiring) or to random nodes (random rewiring).

— *homophilous rewiring* (rate *p*): choose a random edge (*i*, *j*). Choose a random node *k* where *k* ≠ *i*, *S*_*i*_ = *S*_*k*_ and there is no edge (*i*, *k*). Delete edge (*i*, *j*) and add edge (*i*, *k*). If there exists no suitable *k*, do nothing.
— *random rewiring* (rate *q*): choose a random edge (*i*, *j*). Choose a random node *k* such that there is no edge (*i*, *k*). Delete edge (*i*, *j*) and add edge (*i*, *k*). If there is no suitable *k*, do nothing.

The second class of processes changes the states of the nodes: nodes may copy the state of connected nodes or be updated with a random state.

— *symmetric state spread* (rate *r*): choose a random edge (*i*, *j*). Set *S*_*j*_ = *S*_*i*_.
— *innovation* (rate *w*): choose a random node *i* and a random state *s*_*k*_ where ∀*j*, *S*_*j*_ ≠ *s*_*k*_, set *S*_*i*_ = *s*_*k*_.

Note that our implementation of state spread is symmetric in the sense that once an edge is chosen, its endpoints are randomly designated to be source and target. Choosing a random node first which then spreads its state to a neighbouring node would make states with many nodes more likely to spread and invade other state groups; choosing a random node which then copies a neighbouring state, on the other hand, makes states with many nodes more likely to be invaded by other state groups. Our method attempts to avoid these biases.

The rates given for the four processes are to be understood as local (i.e. per-edge or per-node) rates. To obtain global rates, we multiply with the number of edges or nodes, respectively, depending on whether the events are node-based or edge-based. This yields the population-wide rates *mp*, *mq*, *mr* and *nw*.

In simulations run with the state-based processes, we initialize all our nodes with a *null state* to remove any biases from initial conditions. Nodes in that initial state do not actively rewire or spread their state to other nodes until they have been updated with another state. We then wait for a burn-in period until every node has a non-null state before we take measurements on the networks. The distribution of states thus emerges from the model dynamics.

## 3. Results

In the following, we will present our analysis of the dynamics that result from the interplay between the processes outlined above. We will first focus on a scenario of fixed states, where only the two rewiring processes occur, before turning to scenarios where all four processes can happen.

### 3.1. Fixed states

When the state of each node is immutable, the only processes affecting the network are homophilous rewiring, with rate *p*, and random rewiring, with rate *q*. Here, state can be interpreted as a certain property in a simple biological network, or a relatively fixed property of individuals in a social network, such as relative age or a visible trait. We initialize the model by distributing a given number of states randomly among nodes.

**Figure 1.**
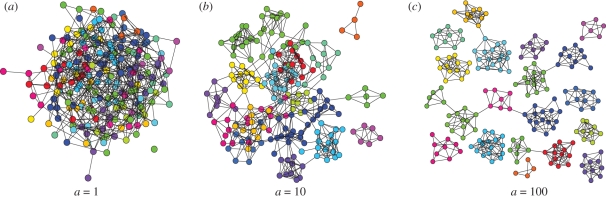
Network snapshots for different values of *a* (where *a* = *p*/*q*) when no state update occurs (i.e. *r* = *w* = 0). Different colours indicate different states. Three classes of stable system behaviour can be distinguished: (*a*) when the rate of random rewiring is high with respect to random rewiring (e.g. *a* = 1), network topology is random; (*b*) when the rate of random rewiring is low (e.g. *a* = 0.01), the network fractures into a set of disconnected, homogeneous components; (*c*) when homophilous and random rewiring are balanced (e.g. *a* = 0.1), densely connected homogeneous state groups are evident, but the network as a whole also remains connected.

When we run the model global network properties such as clustering coefficient, average shortest path length and modularity stabilize in spite of the ongoing dynamics. Generally, three different scenarios of network topology emerge (see figure 1) depending on the distribution of states and the relative fraction of homophilous versus random rewiring events,3.1
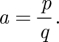
If *a* is small, or most rewiring events connect random nodes, the resulting dynamic networks are of Erdős–Rényi type at any point in time, with the usual characteristics of low clustering, short path lengths and low modularity. If *a* is large, or most rewiring events connect nodes of the same state, groups of nodes sharing the same state form tight communities with only transient connections to the rest of the network. These transient connections, when they come into place, are quickly rewired to again connect nodes of the same state. In that case, while the communities disconnect and reconnect over time, at any specific point in time the network fractures into components of nodes with the same state, with the size of these components depending on the abundance of the corresponding states. These network snapshots possess strong clustering, but since they are disconnected they cannot be associated with meaningful modularity and average path lengths.

Between these two extremes, an intermediate regime of values of *a* exists, where the networks are formed of tightly connected groups of the same state, but there is still enough random rewiring to leave the networks connected at any point in time. In that case, the network snapshots have strong clustering, large modularity and average path lengths.

By considering a population-level analogue of the model described in the previous section, we can use mathematical analysis to predict the modularity of the resulting networks in the intermediate regime. Modularity is a measure of how well a network partition reflects the presence of communities, and is given by [29]3.2
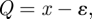
where *x* is the proportion of all edges that are within-module edges—that is, those linking nodes in the same module. The factor *ɛ* = ∑_*i*_ (*d*_*i*_/2*m*)^2^, where *d*_*i*_ is the total degree of nodes in module *i*, corrects for the expected number of links between nodes of the same module if the links were placed at random. A simple algorithm for detecting modules then involves the identification of a network partition that maximizes *Q* [30]. It is worth noting that modularity is not a perfect metric for community structure. It can fail to discriminate between structurally diverse partitions [31], and using modularity to detect communities in large graphs has been demonstrated to miss small communities [32]. These concerns do not preclude the use of modularity for our purposes: our networks are not so large that the resolution limit becomes a serious concern; also, our networks are artificial, and we are more interested in the level of modularity than the identity of modules.

We can take advantage of the fact that homophilous rewiring creates modules of tightly connected nodes of the same state if *a* is large enough. The partition that maximizes *Q* will then be similar to one that identifies nodes of the same state in modules. Therefore, we can use the connections to nodes of the same state and to nodes of a different state as proxies for within-module and between-module connections. In other words, we can interpret *x* to mean the proportion of all edges that are within-state edges, or that link nodes of the same state.

**Figure 2.**
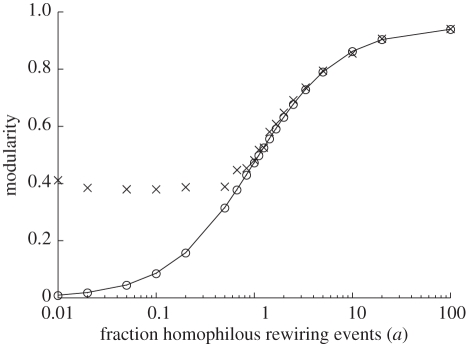
Modularity based on maximal topological modularity as given by the Girvan–Newman algorithm (*Q*_t_) as measured in simulations (crosses), and as given by our algorithm identifying modules based on state (*Q*_s_), as predicted analytically (solid line) and measured in simulations (circles), in terms of the fraction of rewiring events that are homophilous, *a* = *p*/*q*.

If each node is initialized randomly with one of *y* states (0 ≪ *y* ≪ *n*), the value of *ɛ* is given by summing over a Poisson distribution,3.3
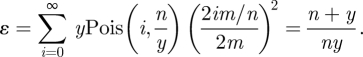
In a similar way *ɛ* can be calculated for other state distributions. Over a period of time where every link is rewired at least once (which is of the order of (*p* + *q*)^−1^), the proportion of within-state edges will converge to *x* ≈ (*p* + *ɛq*)/(*p* + *q*), giving the modularity for the state partition as3.4
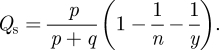
The two processes can thus be used to generate a network that has a partition with modularity given by *Q*_s_. This can be compared with the modularity *Q*_t_ of the partition of the same network that uses topological analysis to maximize modularity (e.g. [9]). When community structure has been introduced by homophilously increasing the numbers of links between nodes of the same state, with all other links placed randomly, it is unlikely that any topological partition that splits up or combines groups of nodes of the same state could achieve a greater level of modularity than that found in the state partition. This intuition is confirmed in figure 2, which shows how the topologically generated partition corresponds to the state partition when the network has topological community structure (*Q*_t_ > 0.4).

### 3.2. Dynamic states

In many systems, such as social systems and neural networks, properties of the nodes in the network can be affected by those they interact with [8,13,33]. For example, in human social systems we tend to form relationships based on an implicit set of criteria such as our interests, political affiliations, socioeconomic status or social norms [20]. However, human adaptability means that the criteria can change—either by copying others we interact with, or by innovating new sets of criteria. To reflect this, we relax the immutability of states and introduce the state spread and innovation processes described above. We may then apply our model to such a system by taking node state to represent a set of criteria shared by many people.

**Figure 3.**
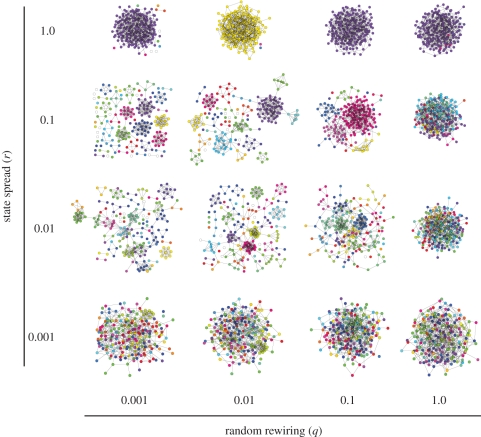
Network snapshots for different rates of state spread (*r*) and random rewiring (*q*) (*p* = 1 and *w* = 0.001). Snapshots were taken at *t* = 5 × 10^6^, to ensure that any transient dynamics had passed. Different colours indicate different states. Again, three classes of stable system behaviour can be distinguished. (i) Random network topologies result not only when the rate of random rewiring is high (*q* = 1), but also when the rate of state spread is either very low or very high. In the former case, the absence of state spread inhibits the organizing tendencies of homophilous rewiring; in the latter case, a single group rapidly establishes itself and dominates the population, in which case homophilous rewiring becomes effectively equivalent to random rewiring. (ii) When the rate of random rewiring is low and there is a moderate level of state spread (e.g. *r* = 0.001; *q* = 0.1), the network fractures into a set of disconnected, homogeneous components. (iii) With intermediate levels of both state spread and random rewiring (e.g. *r* = 0.01; *q* = 0.01), densely connected homogeneous state groups are evident, but the network as a whole also remains connected.

**Figure 4.**
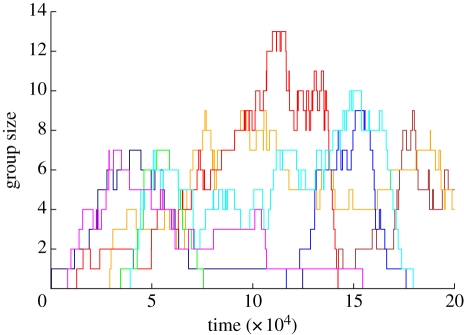
An illustration of the evolution of state groups. This figure plots the size of eight different state groups over 200 000 time steps (*p* = 1; *q* = *r* = *w* = 0.01). The eight state groups shown (of a total of 57 that existed at some point during the simulation run) were each the largest in the population at some point in time.

We find that, under appropriate parameters, the model shows community structure with several concurrent groups, many of which have relatively long lifetimes (figure 3). The sizes of the groups, as well as their composition, are dynamic as nodes join and leave them in the close interplay of state changes and edge rewiring (figure 4). Again, we see that, under a wide range of parameters, some global properties, such as clustering coefficient or network modularity, stabilize as the network keeps evolving. Mathematical analysis (see appendix A.1) also predicts stability of network modularity and gives a good approximation of the corresponding topological network modularity (as with figure 2) when the state spread parameters maintain a moderate number of groups (between *n*/50 and *n*/3).

To capture the mutual feedback between state changes and network rewiring, we introduce two more quantities,3.5
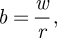
the relative frequency of innovation versus state spread, and3.6
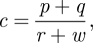
the relative frequency of rewiring versus state update.

Depending on the model parameters, snapshots of the dynamic networks range from random-like graphs with a single or few dominant states to fragmentation into many small tight-knit communities, each of which share the same state (figure 3). In an intermediate regime, highly connected communities emerge, each containing mostly the same state, with some interconnections between those communities, similar to what we observed for fixed states (figure 1). As before, if most rewiring events are homophilous (large *a*), the network tends to have high modularity or even break up into fragments. If, on the other hand, most rewiring events are random (small *a*), network snapshots resemble random graphs. If rewiring happens on time scales faster than state changes (large *c*), we tend to see more modular graphs, whereas if state changes are faster (small *c*), networks are more random. Lastly, the frequency of innovation (regulated by *b*) largely dictates the number of different states concurrently present in the network, with corresponding second-order effects on the distribution of state prevalence and modularity as communities in the network tend to be smaller if there are many concurrent states (see the electronic supplementary material, figure S2).

To characterize the distribution of states at a given moment in time (i.e. how many nodes are in each different state that coexists in a network) we exploit an analogy between our model and the canonical ensemble of statistical physics. This ensemble considers particles in a gas that exchange energy when they collide. In the case of our model, the analogue of particles are the different states, and the equivalent of their energy is the number of nodes that are in that state at a given moment in time. When a state spread event happens, a node in the network changes its state, therefore decreasing the number of nodes in its original state by one and increasing the number of nodes in its new state by one—a process equivalent to the exchange of energy between colliding particles.

**Figure 5.**
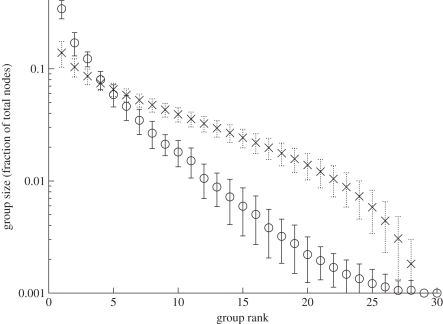
Size distribution of state groups. Shown is the mean size of the *i*th largest group across 20 snapshots from a simulation run (circles; *a* = 100; *b* = 0.001; *c* = 0.3), error bars indicating one standard deviation. Also shown is the distribution as predicted by equation (3.7) (crosses), obtained by sampling from *y* = 28 random numbers summing up to *n* = 1000, using the algorithm of [40], until convergence was obtained. Despite the continually changing composition of state groups in a population (figure 4), distribution of group sizes is relatively stable over time.

**Figure 6.**
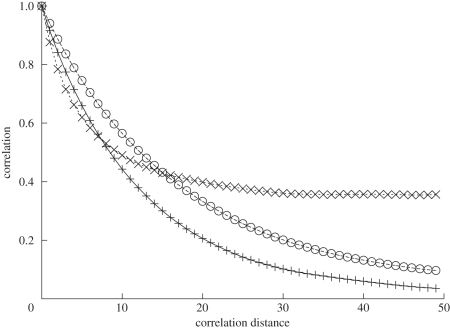
Autocorrelation measures for node and state group properties (*p* = 1.0; *q* = *r* = *w* = 0.01). Node state (pulses) measures the fraction of nodes that are in the same state at time *t* + *d* as they were at time *t*. Node neighbourhood (circles) measures the fraction of node pairs that are neighbours at time *t* + *d* that were also neighbours at time *t*. Group overlap (crosses) measures the relative overlap in group membership between time *t* and time *t* + *d*. Note that all three measures drop rapidly with initial increases in correlation distance; thereafter, some correlation remains at the group level, while node-level correlation drops close to zero.

If we assume such exchanges of nodes between groups of states to occur completely randomly, the probability distribution *P*_*i*_ of groups that have *i* nodes is given by the Boltzmann distribution (see appendix A.2)3.7
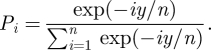
Simulations confirm that the state distribution does indeed stabilize (figure 5). However, while the shape of the distribution remains relatively constant, the identity of groups at a particular rank does not. The ongoing dynamics at the node level causes states to grow and shrink in abundance (figure 6).

The state distribution we observe in simulations is steeper than that given by equation (3.7) (figure 5). The most abundant state tends to contain a greater number of nodes than predicted, and the less abundant ones fewer. This is because large groups of the same state have more edges linking them to other states, and therefore more possibilities to acquire or lose nodes. If, on the other hand, there is only one node left of a given state it can stay in the network for a long time without being selected for an event, or anything happening to it.

**Figure 7.**
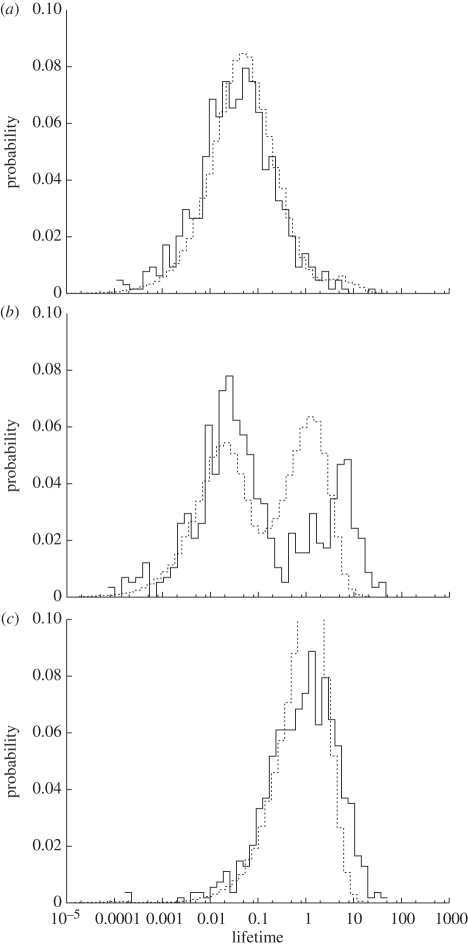
Distribution of the times it takes until a node changes its state (dashed line), and distribution of the total lifetimes of states from first innovation until they go extinct (solid line) for three different sets of parameters representing different relative time scales of state spread and homophilous rewiring: (*a*) fast state spread (*n* = 10^2^; *b* = 10^−3^; *c* = 10^−3^), (*b*) similar time scales (*n* = 10^2^; *b* = 10^−3^; *c* = 10^−1.5^), (*c*) fast rewiring (*n* = 10^1.5^; *b* = 10^−3^; *c* = 10^3^).

In fact, every state that appears in the network via the innovation process will eventually go extinct due to the inherent stochasticity of the model. This becomes clear when we consider the lifetime distribution of states. In figure 7, we compare the distribution of change of states in nodes (i.e. the time it takes until the state of a given node changes) with the distribution of lifetimes of states in all nodes (i.e. the time between a state being introduced through innovation and its extinction) where state spread and homophilous rewiring are much more frequent than the randomizing processes of innovation and random rewiring. When state spread happens on time scales faster than homophilous rewiring, the changes in network structure resulting from rewiring will be too slow to create a modular structure—one dominant group forms and persists for a long time, while most newly innovated states go extinct quickly. Thus the distribution of node state changes and states largely coincide.

If homophilous rewiring and state spread happen with similar frequencies, both distributions are bimodal. The left mode is a reflection of the more than 50 per cent chance of newly innovated states to go extinct before they are spread to a second node (50% for the first spreading event involving the node plus a small chance that another innovation will happen in the same node). Some states, however, become established in the modular network, and the corresponding nodes will form a community and subsequently be protected from invasion as they are surrounded by nodes of the same state. This is why both distributions have another mode at longer lifetimes. Note that the curve representing the lifetime of states has a more pronounced tail because states can survive for a long time even if their composition of nodes change. If homophilous rewiring happens on a much faster time scale than state spread, the distributions again become unimodal. This is because innovations are immediately rewired away from, so that there cannot be rapid extinction.

## 4. Discussion

We have presented a model of dynamic networks in which, over a range of parameters, stable and connected community structure emerges. We have found the presence of such stable community structure to depend largely on the relative frequencies of random to homophilous rewiring. Furthermore, we have provided evidence that a partition of the network according to the state of nodes represents a partition of maximal modularity, and can therefore be used to predict topological modularity. This allowed us to treat modularity analytically, to predict convergent modularity and to quantify its value according to the ratio of random to homophilous rewiring.

The two simple processes of homophily and random rewiring alone can generate community structure reminiscent of that found in the topology of simple, but dynamic, biological networks with units (nodes) having fixed states but dynamic associations (edges). We consider two real-world examples where this is relevant. The first is protein–protein interaction networks where proteins (represented by nodes in our model) often interact (recent or frequent interactions are represented by edges) when they share similar amino acid sequences (represented by states). This homophilous process can explain community structure found in such networks [5,6]. Interestingly, yeast protein interaction data show how communities in the network match well with actual protein complexes [6]. The second example is the Schelling segregation model, which suggests a mechanism for ghetto formation in humans of different ethnic groups [2]. With ethnicity represented by node states, Schelling's model features a rewiring process that only rewires individuals with a high enough proportion of different-state neighbours. This essentially homophilous process leads to a highly modular network. In our model, the introduction of a random rewiring process means that modularity converges to an equilibrium.

When nodes have dynamic states we see how several groups of the same state can exist concurrently in a population with community structure. While the presence of these groups is relatively stable over time, their composition is not: individuals move between groups such that some groups grow, some groups shrink, and others have a roughly constant size, but a continual turnover in members. The behaviour of this model variant is reminiscent of data showing such dynamics in human social and communication networks [34,35] and so may eventually provide insights into how the dynamics on these networks are generated. We characterized the stable group size distribution by comparing it with the Boltzmann distribution, exploiting an analogy of our model to particle collisions in statistical physics. We also compared dynamics at different time scales—the relative time scales of state spread and innovation, as well as the relative time scales of processes affecting states and those affecting the network topology. We have characterized the influence of each of these relative time scales on the behaviour of the network dynamics over a wide range of parameters.

While our model can provide some insight into how endogenous processes produce community structure in real-world networks, there are some limitations to its generality. Communities in real systems can be overlapping [34], and the association between individual nodes and states may be non-exclusive [36], increasing the complexity of both structure and dynamics. Moreover, our model dynamics are biased in that choosing a random edge in the symmetric state spread process increases the frequency with which more highly connected nodes update or spread their states. Other update rules could be argued for, such as degree-based preferential attachment and node-based state spread, each of which would result in different biases.

Future development and validation of our model will require stronger links with data, especially data that are resolved in time. Such data have traditionally been difficult or costly to obtain, though new sources are becoming available, such as online social communities [37,38]. In spite of its limitations, we believe this study to be useful as a systems approach to social modelling [39] and as a baseline to which future models of more specific systems may be compared. We have shown how stable properties can emerge from a highly dynamical system, and focused on modularity, which is a known property of many social and biological systems.

## Appendix A. Mathematical treatment

### A.1. State-based modularity

We can approximate the behaviour of *x* (the proportion of links that connect nodes of the same state) under the four processes in our model by making a few simplifying assumptions.

— *Homophilous rewiring* (rate *mp*): in the random selection of edges, between-state links are selected with probability 1 − *x*, and only in that case does homophilous rewiring take place. Assuming that there is always a node available for rewiring to, the between-state link is replaced with a within-state link. On average, this process thus increases *x* by (1 − *x*)/*m*.
— *Random rewiring* (rate *mq*): if we assume that all edges created through random rewiring are between-state, we only need to consider events rewiring within-state links (as the ones rewiring between-state links do not change *x*). Picking within-state links happens with probability *x*, so this process will on average decrease *x* by *x*/*m*.
— *Symmetric state spread* (rate *mr*): again, if a between-state link is selected (with probability 1 − *x*), it becomes a within-state link. Assuming the node being updated does not have any other links to nodes with its new state, or that the average degree *d* = 2*m*/*n* is small with respect to the number of states currently in the network, it will on average have *xd* within-state links that become between-state links. Including the newly added within-state link, this process on average decreases *x* by (1 − *x*)(*xd* − 1)/*m*.
— *Innovation* (rate *nw*): the updated node will have a new state so all its links will become between-state links. A typical node will have *xd* within-state links, so this process will on average decrease *x* by *xd*/*m*.

We can take all four processes together to give an equation for the temporal evolution of *x*:

A 1

Note that the process of state spread adds a nonlinearity because both the probability of selecting a between-state link, as well as the amount by that the fraction of between-state links is typically changed by state spread, depend on *x* itself.

We derive equilibrium values of *x* by solving  in equation (A 1); these are given by

A 2

Equilibria are stable if and only if

A 3

Substitution of equation (A 3) into equation (A 2) shows that unstable equilibria are only found when the ± term in equation (A 2) is positive, and stable equilibria are found when the ± term in equation (A 2) is negative. Algebraic manipulation can be used to show that unstable equilibria can only be found when . Similarly, stable equilibria are always in the region . This analysis thus shows that for all values of *p*,*q*,*r*,*w*,*d* > 0, there is always a stable equilibrium for *x* in the region .

Further manipulation can be done to show that  will increase for increasing values of *p* (done in this case by comparing  for *p* and *p* + *δ*) and decrease for increasing values of *q*, *w* and *d*. When

will decrease for increasing values of *r*.

The prediction given in equation (A 3) is compared with modularity generated from simulations over a range of parameters in the electronic supplementary material. Both the modularity of the state partition and the maximum modularity from topological analysis were calculated at several time steps (wide enough apart for the network to significantly change) after the burn-in period. The prediction and mean modularities (with standard deviations) are plotted in the electronic supplementary material, figures S1–S3. In the main, the mathematical prediction is good when there is community structure in the network—but there are small differences due to the correction for within-state links expected by a random rewiring of the network (*ɛ*) for the modularity measures. These will decrease as the number of nodes increases. The prediction is also good when the number of states is moderate (between *n*/50 and *n*/3).

### A.2. State distribution

To find the most probable distribution of states, we use an analogy with the distribution of particle energies in an ideal gas. Similarly to the way particles exchange energy in random collisions, the groups of states in our model exchange nodes. We conjecture that the most probable distribution of states *y*_*i*_ can be found by maximizing the number of microstates yielding that distribution (equivalent to minimizing the entropy) under the constraints of constant number of states

A 4

and number of nodes

A 5

The derivation of the most probable distribution follows the same steps as the derivation of the Maxwell–Boltzmann distribution of statistical physics. The number of microstates yielding a distribution *y*_1_, *y*_2_, … , *y*_*n*_ is the number of ways to distribute *y* states among these,

A 6

Maximizing *Ω* (*n*, *y*, {*y*_*i*_}) is equivalent to maximizing

A 7

where we used Stirling's formula, *y*! ≈ *y*^*y*^ e^−*y*^.

We introduce Lagrange multipliers *α* and *β* to impose the constraints of constant number of states and particles:

A 8

and maximize *f*(*y*_*i*_) by solving

A 9

yielding

A 10

as the distribution that maximizes *Ω*(*n*,*y*,{*y*_*i*_}). The first constraint, ∑ *y*_*i*_ = *y*, yields

A 11

so that

A 12

The second constraint, ∑ *iy*_*i*_ = *n*, gives

A 13

To determine *β* analytically, we make a continuum approximation and replace the sums from 1 to *n* by integrals from 0 to infinity. This yields

A 14

and *β* = *y*/*n* via equation (A 13). Putting this back into equation (A 12) and setting *P*_*i*_ = *y*_*i*_/*y* yields equation (3.7).
